# Estimating Healthcare Expenditure Using Parametric Change Point Models

**DOI:** 10.6339/24-jds1157

**Published:** 2024-12-03

**Authors:** Indranil Ghosh, Qi Zheng, Michael E Egger, Maiying Kong

**Affiliations:** 1 Department of Bioinformatics and Biostatistics, School of Public Health and Information Sciences, University of Louisville, Louisville, Kentucky, USA; 2 Department of Biostatistics, Apellis Pharmaceuticals, Waltham, Massachusetts, USA; 3 The Hiram C. Polk, Jr., MD Department of Surgery, School of Medicine, University of Louisville, Louisville, Kentucky, USA; 4 Brown Cancer Center, University of Louisville, Louisville, Kentucky, USA

**Keywords:** changepoint models, healthcare expenditures, pancreatic cancer, phase-based expenditure, SEER-Medicare

## Abstract

Estimating healthcare expenditures is important for policymakers and clinicians. The expenditure of patients facing a life-threatening illness can often be segmented into four distinct phases: diagnosis, treatment, stable, and terminal phases. The diagnosis phase encompasses healthcare expenses incurred prior to the disease diagnosis, attributed to frequent healthcare visits and diagnostic tests. The second phase, following diagnosis, typically witnesses high expenditure due to various treatments, gradually tapering off over time and stabilizing into a stable phase, and eventually to a terminal phase. In this project, we introduce a pre-disease phase preceding the diagnosis phase, serving as a baseline for healthcare expenditure, and thus propose a five-phase to evaluate the healthcare expenditures. We use a piecewise linear model with three population-level change points and 4p subject-level parameters to capture expenditure trajectories and identify transitions between phases, where p is the number of covariates. To estimate the model’s coefficients, we apply generalized estimating equations, while a grid-search approach is used to estimate the change-point parameters by minimizing the residual sum of squares. In our analysis of expenditures for stages I-III pancreatic cancer patients using the SEER-Medicare database, we find that the diagnostic phase begins one month before diagnosis, followed by an initial treatment phase lasting three months. The stable phase continues until eight months before death, at which point the terminal phase begins, marked by a renewed increase in expenditures.

## 1 Introduction

Estimating healthcare expenditures is crucial in the medical field, particularly in understanding the costs associated with specific diseases. These expenditures can vary based on treatments received, patient characteristics, and comorbidities. Moreover, healthcare expenditure may undergo significant changes due to events such as cancer diagnosis, intensive treatment, and death. Given the increasing costs of healthcare delivery, budgetary constraints, and an aging population, it is essential for policy makers and clinicians to understand the trajectory of expenditures for patients diagnosed with a lethal disease such as cancer, which not only has a relatively high incidence rate, but also involves substantial treatment costs (see, e.g., Mihaylova et al., 2011; Wijeysundera et al., 2012).

Although much of the literature focuses on lifetime expenditures associated with specific diseases (Lin et al., 1997; Bang and Tsiatis, 2002; Basu et al., 2011; Li et al., 2016), understanding the patterns and trajectories of expenditures as a disease progresses provides even deeper insights. Recent literature suggests that there are multiple phases involved in the expenditure of a patient diagnosed with cancer (see, e.g., Brown et al., 2002; Wijeysundera et al., 2012; Tramontano et al., 2019). For example, Wijeysundera et al. (2012) and Tramontano et al. (2019) proposed that healthcare care expenditure related to cancer can be categorized into four distinct phases. Understanding these expenditure phases and identifying the points at which they occur is crucial for policymakers and health insurers (Tramontano et al., 2019). Simply averaging expenditures over time may overlook the change points and patterns in expenditures that correspond to different disease phases. Recognizing these change points and patterns is also vital from a research perspective, as it allows investigators to estimate the times at which treatments or disease status change based on expenditure fluctuations.

Despite the importance of these phases for expenditure analysis, many existing studies lack rigorous methods for estimating the corresponding change points. For example, Wijeysundera et al. (2012) and Tramontano et al. (2019) defined expenditure phases and estimated costs using sample means from different sub-cohorts. However, these approaches do not model the change points between phases or account for the influence of patient characteristics and treatment choices on expenditures in each phase. In this project, we propose a statistical framework for estimating change points along with expenditure trajectories influenced by patients’ characteristics and treatment decisions. While change point detection techniques are widely used in economics and meteorology (see, e.g., Reeves et al., 2007; Paulus et al., 2015), their application in medical expenditure analysis is novel.

Another novel aspect of our method is the introduction of an additional phase—the pre-diagnosis phase—to study healthcare expenditures. In evaluating cancer-related costs, Wijeysundera et al. (2012) and Tramontano et al. (2019) categorized cancer-attributable expenditures into four distinct phases: the diagnosis phase, initial treatment phase, stable phase, and terminal phase, each corresponding to different stages of medical care. However, capturing baseline expenditures prior to the diagnosis phase is critical, as it enables the estimation of baseline costs and helps identify the point where expenditures begin to rise. This, in turn, aids in estimating the transition from the pre-disease phase to the diagnosis phase.

**Figure 1:**
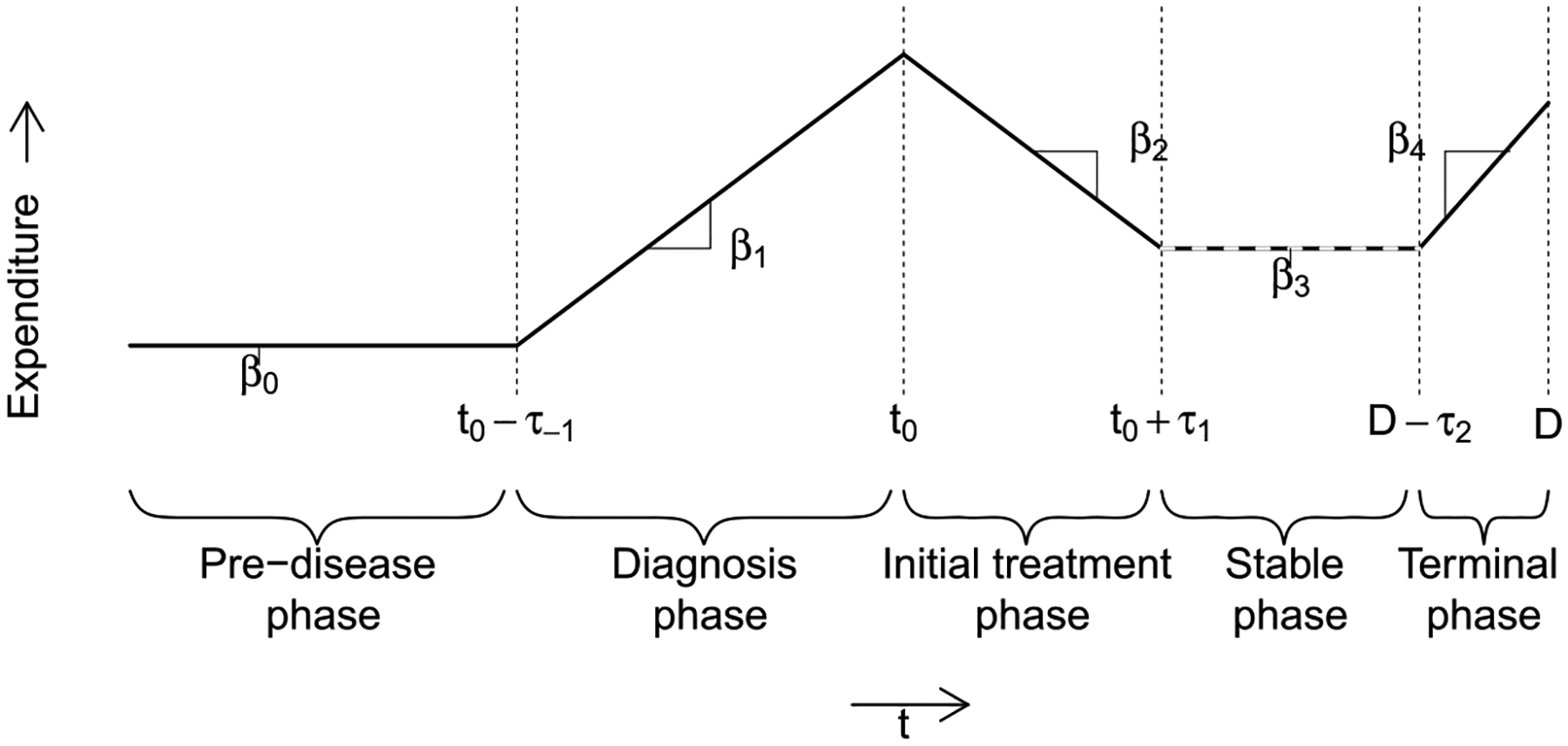
Illustration of expenditure phases and change points.

Our proposed model with five healthcare expenditure phases is illustrated in Figure 1. The five different expenditure phases are defined as follows: (P0) Pre-disease phase, this phase occurs before diagnosis $\left(t_{0}\right)$, where patients may not be aware of any disease or may not have any health conditions until a certain time point (say, $t_{0}-\tau_{-1}$) before diagnosis. The baseline expenditure parameter in this phase is denoted by $\beta_{0}$, representing the patient’s expenditure in good health, serving as a baseline for recognizing subsequent changes. (P1) Diagnosis phase, this phase begins from the change point before diagnosis $\left(t_{0}-\tau_{-1}\right)$ and extends to the time of diagnosis of the disease at $t_{0}$. During this period, extensive diagnostic tests and procedures are typically performed, resulting in higher medical expenditure. The parameter associated with this phase is denoted by $\beta_{1}$, capturing the change in monthly expenditure during the diagnosis phase. (P2) Initial treatment phase: this phase starts from the diagnosis time $t_{0}$ and continues until the end of intensive treatment at time $t_{0}+\tau_{1}$. Expenditure during this phase tends to decrease over time until it reaches a relatively stable level. The expenditure parameter associated with this phase is denoted by $\beta_{2}$, capturing the change in monthly expenditure during the initial treatment phase. (P3) Stable phase: this phase lies between the end of the initial treatment phase at time $t_{0}+\tau_{1}$ and the beginning of the terminal phase at time ($D-\tau_{2}$), where patients become severely ill again, that is $\tau_{2}$ months before the end of life at time D. The expenditure parameter associated with this phase is denoted by $\beta_{3}$, representing the average monthly expenditure for the patient during the stable phase. (P4) Terminal phase: this phase spans from becoming severely ill after the stable phase at $\left(D-\tau_{2}\right)$ to the end of life at time D. The expenditure parameter associated with this phase is denoted as $\beta_{4}$, capturing the change in monthly expenditure during the terminal phase.

We use a piecewise linear model with three population-level change points $\tau ={\left(\tau_{-1},\tau_{1},\tau_{2}\right)}^{T}$ and 4p subject-level parameters to capture expenditure trajectories and identify transitions between phases, where p is the number of covariates. It is widely recognized that medical expenditures are often influenced by the treatment received and patient comorbidities (Austin, 2011). To account for patient-level characteristics and treatment effects, we allow the expenditure parameters $\beta ={\left(\beta_{0},\beta_{1},\beta_{2},\beta_{3},\beta_{4}\right)}^{T}$ to be patient-specific and model them based on individual patient’s characteristics, enabling a more comprehensive understanding of how these characteristics and treatment choices influence healthcare expenditure trajectories across different phases of disease progression and treatment.

The remainder of the paper is structured as follows. In Section 2, we present detailed information on the proposed method. Section 3 is dedicated to applying the proposed method to estimate the expenditure trajectory for pancreatic cancer patients with stages I–III in the SEER-Medicare 2005–2014 database. The final section, Section 4, is reserved for discussion.

## 2 The Proposed Model for Change Point Detection and Expenditure Trajectory Estimation

### 2.1 A Simple Change Point Model

Without loss of generality, we set $t_{0}$ as time 0, since we can always align and standardize the patients’ expenditures from the time of diagnosis. Let C(t) denote the expenditure during month t, and let D denote the time of death since diagnosis. We assume that everyone in the cohort has survived the diagnosis, with D>0. Within the five-phase framework as illustrated in Figure 1 and based on established change points models in the literature (see, e.g., Reeves et al., 2007; Paulus et al., 2015), the expected expenditure trajectory during the pre-disease phase and diagnosis phase can be captured by a 3-parameter model $E[C(t)]=\left[\beta_{0}+\beta_{1}{\left(t-\tau_{-1}\right)}^{+}\right]I_{\left(t<0\right)}$, where $A^{+}=A$ if A>0 and 0 otherwise for a generic quantity A, and $I_{\left(\cdot \right)}$ is the indicator function such that $I_{\left(A\right)}=1$ if a generic event A is true and 0 otherwise. The expected expenditure profile from diagnosis to death can be captured by a 5-parameter model: $E[C(t)]=\left[\beta_{3}+\beta_{2}{\left(\tau_{1}-t\right)}^{+}\right]I_{\left(t⩾0\right)}+\beta_{4}{\left(t-\left(D-\tau_{2}\right)\right)}^{+}$. Thus, the proposed piece-wise linear model with three change points can be written as:

(1) $$C(t)=\left[\beta_{0}+\beta_{1}{\left(t-\tau_{-1}\right)}^{+}\right]I_{\left(t<0\right)}+\left[\beta_{3}+\beta_{2}{\left(\tau_{1}-t\right)}^{+}\right]I_{\left(t⩾0\right)}+\beta_{4}{\left(t-\left(D-\tau_{2}\right)\right)}^{+}+\epsilon \left(t\right),$$

where ϵ(t) is a random variable with Gaussian process of autoregressive model of order one (AR(1)) and zero means. Since the expenditure function in practice is often continuous, we impose the constraint $\beta_{0}+\beta_{1}\left(0-\tau_{-1}\right)=\beta_{3}+\beta_{2}\left(\tau_{1}-0\right)$ to ensure that E[C(t)] is continuous at 0, where the 3-parameter model for the first two phases and the 5-parameter model for the last three phases meet. Thus, by replacing $\beta_{3}=\beta_{0}-\beta_{1}\tau_{-1}-\beta_{2}\tau_{1}$ in C(t), the expectation of the expenditure function in equation (1) can be written as:

(2) $$E[C(t)]=\left[\beta_{0}+\beta_{1}{\left(t-\tau_{-1}\right)}^{+}\right]I_{\left(t<0\right)}+\left[\beta_{0}-\beta_{1}\tau_{-1}-\beta_{2}\tau_{1}+\beta_{2}{\left(\tau_{1}-t\right)}^{+}\right]I_{\left(t⩾0\right)}+\beta_{4}{\left(t-\left(D-\tau_{2}\right)\right)}^{+}\;=\beta_{0}+\beta_{1}\left[{\left(t-\tau_{-1}\right)}^{+}I_{\left(t<0\right)}-\tau_{-1}I_{\left(t⩾0\right)}\right]+\beta_{2}\left[\left(-\tau_{1}+{\left(\tau_{1}-t\right)}^{+}\right)I_{\left(t⩾0\right)}\right]+\beta_{4}{\left(t-\left(D-\tau_{2}\right)\right)}^{+}=Z^{⊤}\beta ,$$

where $Z={\left(1,{\left(t-\tau_{-1}\right)}^{+}I_{\left(t<0\right)}-\tau_{-1}I_{\left(t⩾0\right)},\left(-\tau_{1}+{\left(\tau_{1}-t\right)}^{+}\right)I_{\left(t⩾0\right)},{\left(t-\left(D-\tau_{2}\right)\right)}^{+}\right)}^{⊤},\beta ={\left(\beta_{0},\beta_{1},\beta_{2},\beta_{4}\right)}^{⊤}$, and $\tau ={\left(\tau_{-1},\tau_{1},\tau_{2}\right)}^{⊤}$. Both β and τ are unknown and must be estimated. Additionally, $\beta_{3}$ can be estimated from the relationship $\beta_{3}=\beta_{0}-\beta_{1}\tau_{-1}-\beta_{2}\tau_{1}$. Here $\beta_{0}$ and $\beta_{3}$ represent the expenditure per time unit in the pre-disease phase and stable phase, respectively; $\beta_{1}$ and $\beta_{4}$ capture the rate of expenditure increase per time unit in the diagnosis phase and terminal phase, respectively; and $\beta_{2}$ captures the rate of the expenditure decrease per time unit in the initial treatment phase. $\tau_{-1}$ represents the time units prior to diagnosis marking the entry into the diagnosis phase, $\tau_{1}$ represents the time units post-diagnosis to the end of the initial treatment phase, and $\tau_{2}$ represents the time units before death indicating the start of the terminal phase.

Note that the third term in equations (1) and (2), $\beta_{4}{\left(t-\left(D-\tau_{2}\right)\right)}^{+}$, does not incorporate a time indicator variable, indicating that the terminal phase is consistently present and lasts for $\tau_{2}$ months. In situations where the death time D is shorter than the combined durations of the initial treatment phase and the terminal phase, i.e., $D<\tau_{1}+\tau_{2}$, equation (2) still holds, but there is no stable phase in between. If $D<\tau_{2}$, the expenditures prior to diagnosis are a mixture of the diagnostic and terminal phases, while expenditures post-diagnosis are a combination of the initial treatment and terminal phases. If $\tau_{2}⩽D<\tau_{1}+\tau_{2}$, the post-diagnosis expenditures include the initial treatment phase, followed by a combination of the initial treatment phase and the terminal phase.

The expenditure profile often depends on patient characteristics such as age, comorbid conditions, and treatment received (Austin, 2011). We propose a model in which the regression parameters β are patient-specific and depend on individual patient variables, while the change point parameters remain population-specific. This approach allows us to understand how individual patient characteristics influence healthcare expenditure trajectories over time, while still accounting for common trends observed across the population.

### 2.2 The Proposed Patient-Level Change Point Expenditure Models

Let (X,𝒯,C,δ) denote the random variable observed for a patient. X represents a vector of p time-invariant covariates of the patient, including patients’ characteristics, medical history, and treatment information. $𝒯={\left(t_{-a},\ldots ,t_{0},t_{1},\ldots ,t_{b}\right)}^{⊤}$ denotes the vector of time points at which medical expenditures $C={\left(C_{-a},\ldots ,C_{0},C_{1},\ldots ,C_{b}\right)}^{⊤}$ are recorded, where we set $t_{0}=0.\delta$ is an indicator variable that specifies whether the patient died at the last observed time point $t_{b}$, with δ=1 indicating death at time $t_{b}$.

The proposed model (2) incorporates each patient’s survival time, D. For patients who died during the study period, the observed survival time is used in equation (2). For censored patients, the median of the residual lifetime is estimated based on a working model such as the accelerated failure time (AFT) model. That is, if δ=1, the survival time D is equal to the last observed time point $t_{b}$. If δ=0, the survival time D is estimated as the sum of the predicted median of the residual lifetime and the censoring time. A terminal expenditure phase, as described in equation (2), occurs for a patient with censored death (i.e., δ=0) if the last observed time point $t_{b}$ satisfies the condition $t_{b}>\overset{ˆ}{D}-\tau_{2}$, where $\overset{ˆ}{D}$ is the sum of the predicted median of the residual lifetime and the censoring time.

Let ${\left(X_{i},{𝒯}_{i},C_{i},\delta_{i}\right)}_{i=1}^{N}$ represent the observed data for N patients in the study. The expected expenditure trajectory for each patient is assumed to follow the pattern outlined in Figure 1. We propose that the parameters β in equation (2) are patient-specific, denoted as $\beta_{i}$ for the ith patient (i=1,…,N), while the change point parameters τ remain population-specific. The expenditure for the ith patient at time $t_{ij}$ can be written as: $C_{ij}=Z_{ij}^{⊤}\beta_{i}+\epsilon_{ij}$, where $C_{ij}=C\left(t_{ij}\right),Z_{ij}={\left(1,{\left(t_{ij}-\tau_{-1}\right)}^{+}I_{\left(t_{ij}<0\right)}-\tau_{-1}I_{\left(t_{ij}⩾0\right)},\left(-\tau_{1}+{\left(\tau_{1}-t_{ij}\right)}^{+}\right)I_{\left(t_{ij}⩾0\right)},{\left(t_{ij}-\left(D_{i}-\tau_{2}\right)\right)}^{+}\right)}^{⊤}$, and $\beta_{i}={\left(\beta_{i0},\beta_{i1},\beta_{i2},\beta_{i4}\right)}^{⊤}$. We model $\beta_{i}$ as a linear function of the patient covariates $X_{i}={\left(X_{i1},X_{i2},\ldots ,X_{ip}\right)}^{⊤}$. That is

$$\beta_{i}=\left[\begin{array}{llll} \gamma_{01} & \gamma_{02} & \cdots & \gamma_{0p}\\ \gamma_{11} & \gamma_{12} & \cdots & \gamma_{1p}\\ \gamma_{21} & \gamma_{22} & \cdots & \gamma_{2p}\\ \gamma_{41} & \gamma_{42} & \cdots & \gamma_{4p} \end{array}\right]X_{i}≜\Gamma X_{i},$$

where $\Gamma \in R^{4\times p}$ is the parameter matrix to be estimated. Once we have Γ estimated (denoted as $\overset{ˆ}{\Gamma }$), we can gauge the contribution of each covariate on the expenditure profile in each phase. Further, we can also predict the patient-level expenditure profile for the ith patient with covariate $X_{i}$ using the relationship ${\overset{ˆ}{\beta }}_{i}=\overset{ˆ}{\Gamma }X_{i}$. Note that

(3) $$C_{ij}=Z_{ij}^{⊤}{\;\beta }_{i}+\epsilon_{ij}=Z_{ij}^{⊤}\Gamma X_{i}+\epsilon_{ij}.$$

To estimate Γ, we apply Roth’s Columns Lemma (Roth, 1934), which is popularly known as the “vec trick”, to equation (3):

$$C_{ij}=\left[{X_{i}}^{⊤}⊗Z_{ij}^{⊤}\right]vec\left(\Gamma \right)+\epsilon_{ij},$$

where

$$\left[{X_{i}}^{⊤}⊗Z_{ij}^{⊤}\right]=\left[X_{i1}Z_{ij}^{⊤},X_{i2}Z_{ij}^{⊤},\ldots ,X_{ip}Z_{ij}^{⊤}\right]\in R^{4p}$$

and $vec(\Gamma )=\left(\gamma_{01},\gamma_{11},\gamma_{21},\gamma_{41},\gamma_{02},\gamma_{12},\gamma_{22},\gamma_{42},\ldots ,\gamma_{0p},\gamma_{1p},\gamma_{2p},\gamma_{4p}\right)\in R^{4p}$. The expenditures of the ith patient at the time sequence ${𝒯}_{i}={\left(t_{i\left(-a_{i}\right)},\ldots ,t_{i0},t_{i1},\ldots ,t_{ib_{i}}\right)}^{⊤}$ are

(4) $$C_{i}=\left[{X_{i}}^{⊤}⊗Z_{i}^{⊤}\right]vec\left(\Gamma \right)+\epsilon_{i},$$

where

$$Z_{i}^{⊤}=\left[\begin{array}{c} Z_{i\left(-a_{i}\right)}^{⊤}\\ \vdots \\ Z_{i(0)}^{⊤}\\ \vdots \\ Z_{ib_{i}}^{⊤} \end{array}\right],\;{X_{i}}^{⊤}⊗Z_{i}^{⊤}=\left[\begin{array}{c} X_{i1}Z_{i\left(-a_{i}\right)}^{⊤},X_{i2}Z_{i\left(-a_{i}\right)}^{⊤},\ldots ,X_{ip}Z_{i\left(-a_{i}\right)}^{⊤}\\ \vdots \\ X_{i1}Z_{i\left(0\right)}^{⊤},X_{i2}Z_{i\left(0\right)}^{⊤},\ldots ,X_{ip}Z_{i\left(0\right)}^{⊤}\\ \vdots \\ X_{i1}Z_{ib_{i}}^{⊤},X_{i2}Z_{ib_{i}}^{⊤},\ldots ,X_{ip}Z_{ib_{i}}^{⊤} \end{array}\right],$$

$Z_{i}^{⊤}\in R^{\left(a_{i}+b_{i}+1\right)\times 4},X_{i}^{⊤}⊗Z_{i}^{⊤}\in R^{\left(a_{i}+b_{i}+1\right)\times 4p}$, and each $\epsilon_{i}$ follows a multivariate normal distribution $\text{MVN}\left(0,\sigma^{2}R_{i}\right)$ with $R_{i}$ being an auto-regressive correlation matrix with a common first-order correlation coefficient ρ to capture the possible correlations among observed expenditures over time for the ith patient. To evaluate Γ and the change points, we expand equation (4) for the entire sample as:

(5) $$C=\left[\begin{array}{c} {X_{1}}^{⊤}⊗Z_{1}^{⊤}\\ \vdots \\ {X_{i}}^{⊤}⊗Z_{i}^{⊤}\\ \vdots \\ {X_{N}}^{⊤}⊗Z_{N}^{⊤} \end{array}\right]vec(\Gamma )+\epsilon ,$$

where $C={\left(C_{1},\ldots ,C_{i},\ldots ,C_{N}\right)}^{⊤}\in R^{\sum_{i=1}^{N}\left(a_{i}+b_{i}+1\right)\times 1}$, and $\epsilon ={\left(\epsilon_{1},\ldots ,\epsilon_{N}\right)}^{⊤}$ is the vector of random noises with $\epsilon_{i}$ and $\epsilon_{i^{\prime }}$ being mutually independent. Given the potentially large number of parameters (i.e., Γ) to be estimated in the model, there is a risk of overfitting. To address this, we apply penalized generalized estimating equations (PGEE) (Wang et al., 2012) to estimate the parameters Γ. PGEE is effective for simultaneous variable selection and estimation, particularly when the number of covariates in the model is large.

### 2.3 The Estimation Procedure

The key parameters in the proposed change point model are the parameter matrix

$$\Gamma =\left[\begin{array}{llll} \gamma_{01} & \gamma_{02} & \cdots & \gamma_{0p}\\ \gamma_{11} & \gamma_{12} & \cdots & \gamma_{1p}\\ \gamma_{21} & \gamma_{22} & \cdots & \gamma_{2p}\\ \gamma_{41} & \gamma_{42} & \cdots & \gamma_{4p} \end{array}\right]$$

and the change points $\tau =\left(\tau_{-1},\tau_{1},\tau_{2}\right)$.

Subsequently, we can obtain the patient-level expenditure profile using the relationship $\beta_{i}={\left(\beta_{i0},\beta_{i1},\beta_{i2},\beta_{i4}\right)}^{⊤}=\Gamma X_{i}(i=1,\ldots ,N)$ and $\beta_{i3}=\beta_{i0}-\beta_{i1}\tau_{-1}-\beta_{i2}\tau_{1}$. To estimate Γ and τ, we first need to estimate the time of death for censored patients using a survival model. We propose using the AFT model to predict the median of the residual lifetime, after which the survival time is estimated as the sum of the predicted residual lifetime and the censoring time for patients with $\delta_{i}=0$. The median of the residual lifetime for patients with $\delta_{i}=0$ is estimated using the AFT model and the covariates $X_{i}(i=1,\ldots ,N)$. Next, we specify how to estimate the change points, τ, which are population-specific parameters in our proposed model. According to existing literature for cancer patients (see, e.g. Tramontano et al., 2019), $\tau_{-1},\tau_{1}$, and $\tau_{2}$ are often considered as 2 months prior to diagnosis, 6 months after diagnosis, and 6 months before death, respectively. We expand the possible set of values for each change point parameter. Namely, we assume that $\tau_{-1}$ is within a grid of possible values (−6, −5, −4, −3, −2, −1), $\tau_{1}$ is within (1, 2, 3, 4, 5, 6) and $\tau_{2}$ is within (5, 6, 7, 8, 9, 10). Thus we totally have 6 × 6 × 6 = 216 possible combinations for the value of τ. For each combination of τ, we estimate Γ by using the PGEE method (Wang et al., 2012; Inan and Wang, 2017). We then calculate the residual mean square errors (RMSE) for model (5) based on the estimated $\overset{ˆ}{\Gamma }$ for a given τ. The final estimated $\overset{ˆ}{\tau }$ is the one that minimizes the RMSE. The $\overset{ˆ}{\Gamma }$ is the estimate corresponding to the selected $\overset{ˆ}{\tau }$.

To make inferences on the change points’ parameters $\tau ={\left(\tau_{-1},\tau_{1},\tau_{2}\right)}^{T}$, we use the non-parametric bootstrap resampling scheme to evaluate their accuracy and distribution. We obtain B bootstrap samples (say B=1000) from the observed data and then repeat the same estimation procedure for each bootstrap sample. Subsequently, we obtain the distribution of $\overset{ˆ}{\tau }$, which provides insight into the accuracy of the optimal selection for τ. Additionally, we provide the estimated $\overset{ˆ}{\Gamma }$ matrix along with its standard errors to evaluate the impact of different covariates on healthcare expenditures in different phases. We further estimate the expenditure profile for each patient through the parameter $\beta_{i}$. These expenditure profiles can be summarized at the population level or within subgroups of interest by calculating the average of the estimated subject-level parameters in each subgroup.

## 3 Case Study

We applied our proposed method to SEER-Medicare 2005–2014 pancreatic cancer data to investigate healthcare expenditure patterns over time and their association with various covariates. Our primary objective was to estimate population-specific change points and the parameter matrix Γ, along with patient-level expenditure trajectories. This case study delved into the expenditure patterns related to patients with stage I–III pancreatic cancers throughout their diagnoses and treatments across different phases.

This study utilized the 2005–2014 SEER cancer file along with Parts A and B claims files. The study cohort comprised patients diagnosed with pancreatic cancer at the primary site, with specified histology and behavioral codes in the SEER cancer file between March 2006 and December 2013. Eligibility required patients to receive at least one treatment within six months of diagnosis. All cohort members were continuously enrolled in Medicare Parts A and B, without HMO coverage, from 14 months prior to diagnosis until December 2014 or death, whichever occurred first. In cases of multiple diagnoses, the initial occurrence was considered. Comorbidities were assessed using the NCI comorbidity index, based on data from the year preceding the pancreatic cancer diagnosis. This index, developed by Klabunde et al. (2000), is tailored specifically for cancer and excludes solid tumors, leukemia, and lymphomas as comorbid conditions. The NCI comorbidity index was calculated using the 2014 NCI SAS Macro (NCI, 2014), leveraging data from the SEER cancer file, inpatient file (Medpar), outpatient file (Outpat) and carrier claims file (NCH). Covariates obtained from the SEER cancer file included demographic variables (race, age, and sex), geographical variables (Metro vs. non-Metro), and cancer-specific variables (grade 1–4; stages I & II vs III). Treatment assignments were categorized as chemotherapy or surgery, based on the first intervention administered post-diagnosis. Our study specifically targeted patients aged 65 and older, diagnosed with stage I–III pancreatic cancer, who received either surgery or chemotherapy. After applying these inclusion and exclusion criteria, our sample consisted of 2,899 patients. Of these, 2,277 patients passed away during the study period, while 622 survived until the end of the study in December 2014.

All expenditures from Medpar, Outpat, and NCH files were recorded as observed expenditures, reflecting payments made by Medicare. These expenditures were adjusted to 2014 rates using the Consumer Price Index (CPI) data (US Department of Labor Bureau of Labor Statistic, 2021). For each patient, the time of diagnosis was set as the origin ($t_{0}=0$), and monthly expenditures were calculated by summing all expenditures incurred during that month. To adequately capture the pre-disease phase expenditure, we limited our observation period to 14 months prior to diagnosis. This ensured sufficient data to delineate the expenditure trajectory during this phase. For a comprehensive analysis of expenditures, we followed patients over time until death or the end of the study period in December 2014, whichever occurred first. The diagnostic window was set from March 2006 to December 2013 to ensure (1) at least 14 months to investigate the expenditure patterns prior to diagnosis, encompassing the pre-disease and diagnostic phases, and (2) a minimum of one year of follow-up to assess post-diagnosis Medicare expenditure patterns for the cohort.

**Figure 2:**
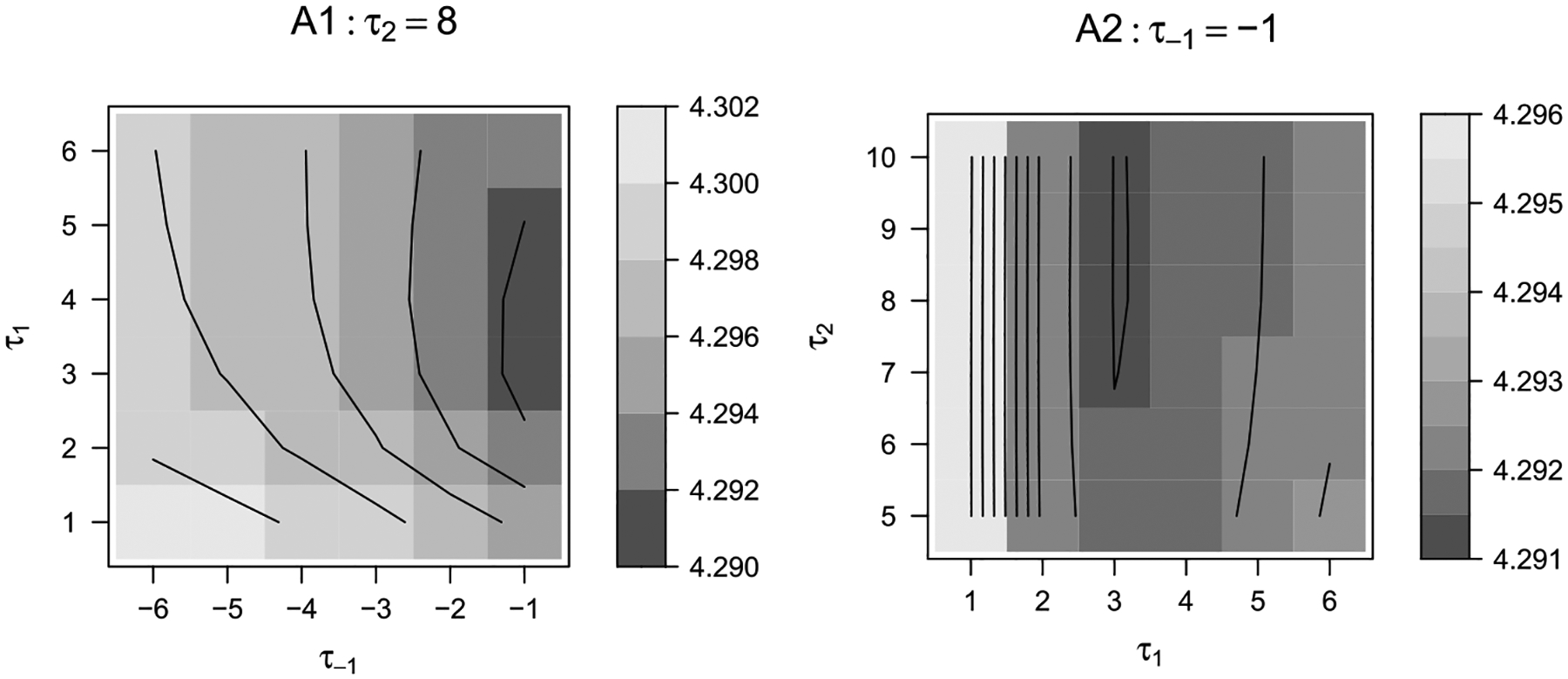
The contour plot of RMSE for ${\overset{ˆ}{\tau }}_{1}$ versus ${\overset{ˆ}{\tau }}_{-1}$ with ${\overset{ˆ}{\tau }}_{2}$ fixed at the optimal value of 8 (Panel A1), and the contour plot of RMSE for ${\overset{ˆ}{\tau }}_{2}$ versus ${\overset{ˆ}{\tau }}_{1}$ with ${\overset{ˆ}{\tau }}_{-1}$ fixed at the optimal value of −1.

As healthcare expenditure data are often highly skewed, we transformed the expenditures into a logarithmic scale to fit our proposed model, following previous literature (see, e.g., Manning and Mullahy, 2001; Başer et al., 2004). We set 6 possible values for each change point parameter: $\tau_{-1},\tau_{1}$ and $\tau_{2}$ range from −6 to −1, 1 to 6, and 5 to 10, respectively. In this case study, the optimal choice of $\tau ,\overset{ˆ}{\tau }$, which minimized the RMSE of our proposed model, was obtained as (−1, 3, 8). Figure 2 Panel A1 illustrates the contour plot of RMSE for different choices of $\tau_{-1}$ and $\tau_{1}$, with $\tau_{2}$ fixed at the optimal value 8. Figure 2 Panel A2 presents the contour plot of RMSE for different choices of $\tau_{1}$ and $\tau_{2}$, with the optimal choice of $\tau_{-1}$ set at −1. From Figure 2, it is evident that our optimal selection of $\overset{ˆ}{\tau }=(-1,3,8)$ minimized the RMSE of the model among all 216 possible values of τ. We further estimated the distribution of $\overset{ˆ}{\tau }$ by performing 1000 bootstrap samplings. The relative frequencies of the selected change points τ are shown in Table 1. It is clear that each component of the selected $\overset{ˆ}{\tau }=\left({\overset{ˆ}{\tau }}_{-1},{\overset{ˆ}{\tau }}_{1},{\overset{ˆ}{\tau }}_{2}\right)=(-1,3,8)$ was the mode of its distribution.

With the change point parameters fixed at $\overset{ˆ}{\tau }$ and the estimated time of death, the design matrix $X_{i}$ in the model (5) was determined. We then estimated the regression parameter matrix $\overset{ˆ}{\Gamma }$ using the PGEE method. The estimated parameter matrix $\overset{ˆ}{\Gamma }$ and their standard errors are shown in Table 2. From the $\overset{ˆ}{\Gamma }$ matrix, we calculated the expenditure profile parameters $\overset{ˆ}{\beta }={\left({\overset{ˆ}{\beta }}_{0},{\overset{ˆ}{\beta }}_{1},{\overset{ˆ}{\beta }}_{2},{\overset{ˆ}{\beta }}_{4}\right)}^{T}=\overset{ˆ}{\Gamma }X$, and computed ${\overset{ˆ}{\beta }}_{3}={\overset{ˆ}{\beta }}_{0}-{\overset{ˆ}{\beta }}_{1}{\overset{ˆ}{\tau }}_{-1}-{\overset{ˆ}{\beta }}_{2}{\overset{ˆ}{\tau }}_{1}$, which captures the expenditure during the stable phase. The estimated $\overset{ˆ}{\Gamma }$, along with their standard errors (SE) and p-values, are presented in Table 2.

Based on the estimated regression coefficients $\overset{ˆ}{\Gamma }$ for $\beta_{0}$ (i.e., the first row block) in Table 2, it is evident that for each unit increase in the NCI comorbidity index, the baseline monthly expenditure rises by $262.1. The second row of $\overset{ˆ}{\Gamma }$ for $\beta_{1}$ indicates both the NCI comorbidity index and surgery (compared to Chemotherapy) significantly increase diagnosis expenditure. Factors such as the NCI index, tumour grade, sex, stage of cancer, and the type of treatment provided significantly impact expenditure profiles. For example, females incur higher expenditure during pre-disease phase but lower expenditure in the diagnosis phase and initial treatment phase. Surgery emerges as a major driver of expenditures in the diagnosis and treatment phases. Earlystage cancers are more likely to be treated surgically, resulting in higher initial expenditures. In contrast, patients with stage 3 or 4 pancreatic cancer are typically deemed unresectable and are treated with chemotherapy alone in the initial treatment phase, thereby incurring no surgical expenses. Thus, we can use the $\overset{ˆ}{\Gamma }$ values along with their SEs in Table 2 to infer the effects of the covariates on expenditure profiles across different phases.

We further summarized the observed and estimated monthly expenditures in terms of mean expenditure and standard deviation (SD) for the study cohort during different expenditure phases in Table 3. It is evident that the monthly expenditure during the diagnosis phase was the highest, followed by the initial treatment phase, and then the terminal phase. The expenditure during the stable phase was higher than in the pre-disease phase. From Table 3, we also conclude that the estimated expenditures closely align with the observed expenditures in each phase.

**Figure 3:**
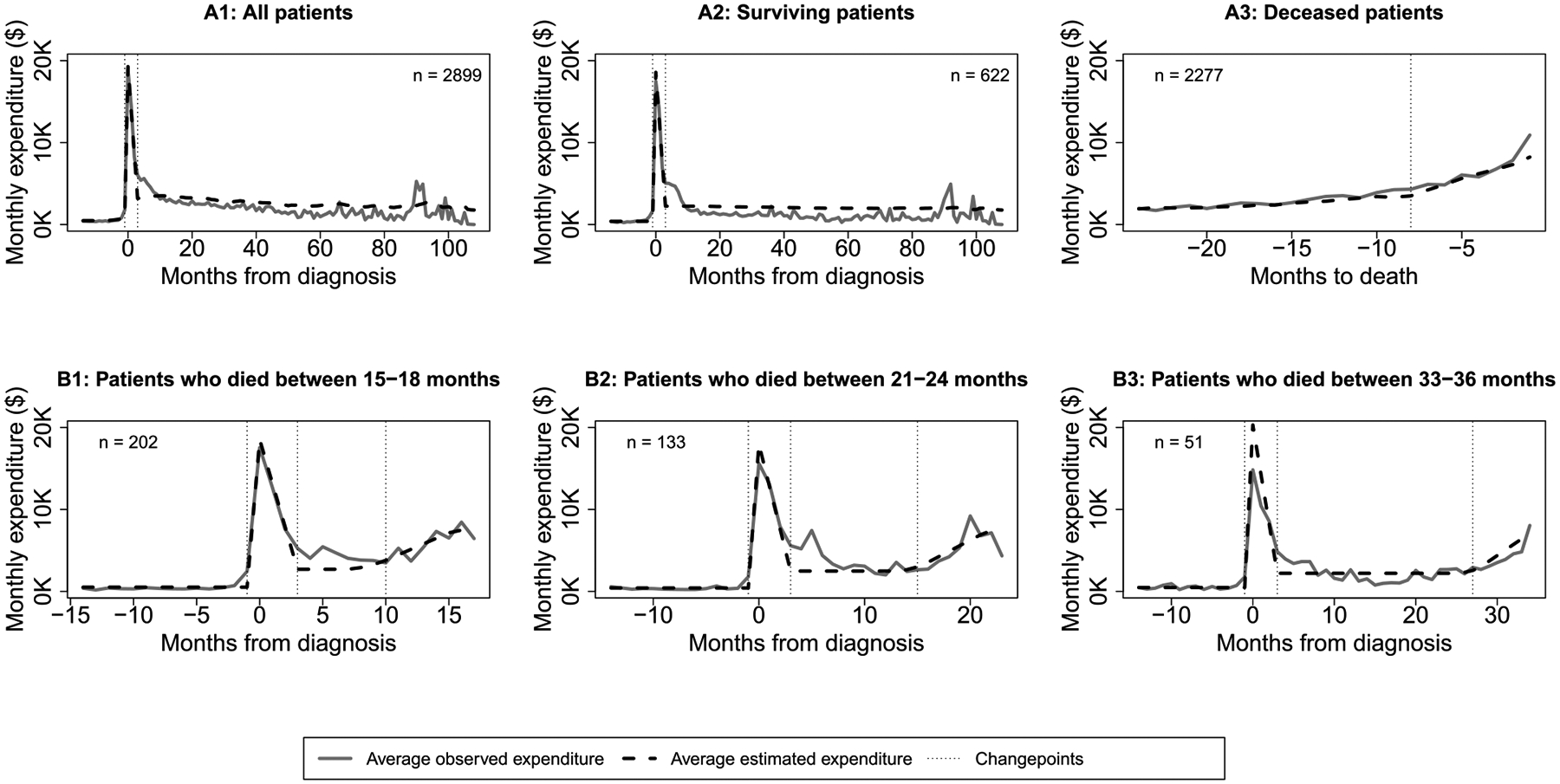
Average observed and estimated monthly expenditure along with change points for different sub-cohorts of pancreatic cancer patients using the proposed parametric change point approach.

Finally, Figure 3 provides the average observed monthly expenditure (solid line) and average predicted monthly expenditure (dashed lines) for different selected sub-cohorts, along with the estimated change points at $\overset{ˆ}{\tau }=\left({\overset{ˆ}{\tau }}_{-1},{\overset{ˆ}{\tau }}_{1},{\overset{ˆ}{\tau }}_{2}\right)=(-1,3,8)$ (dotted lines). Figure 3 Panel A1 shows the expenditure and its estimation for the entire cohort, while Panel A2 focuses on patients who survived during the study period. Panels B1–B3 present similar results but for different sub-cohorts based on their survival time since diagnosis. Panel B1 includes patients who died between 15–18 months after diagnosis, Panel B2 includes those who died between 21–24 months after diagnosis, and Panel B3 features patients who died between 33–36 months after diagnosis. Note that all plots are aligned to the time of diagnosis as the origin (at time 0) on the x-axis. Therefore, we cannot display $\tau_{2}$ in Panels A1 and A2, as the time varies across patients. Panels B1–B3 provide a similar representation but with a narrower range of survival times, with the x-axis extending to the longest survival time in each subcohort. However, these representations again do not accurately depict $\tau_{2}$, which captures the beginning of the terminal phase prior to death. Since the time of death varied for each patient, the best representation of $\tau_{2}$ would align the x-axis with respect to the time of death. Panel A3 of Figure 3 summarizes the expenditure aligned with death, illustrating the role of $\tau_{2}$. Here, the x-axis is set to the time of death as the origin and represents months prior to the time of death. The optimal value $\tau_{2}$ is plotted at −8 months, clearly indicating an upward trend in expenditure starting 8 months before death, thus confirming that the estimated $\tau_{2}$ effectively captured the change point.

**Figure 4:**
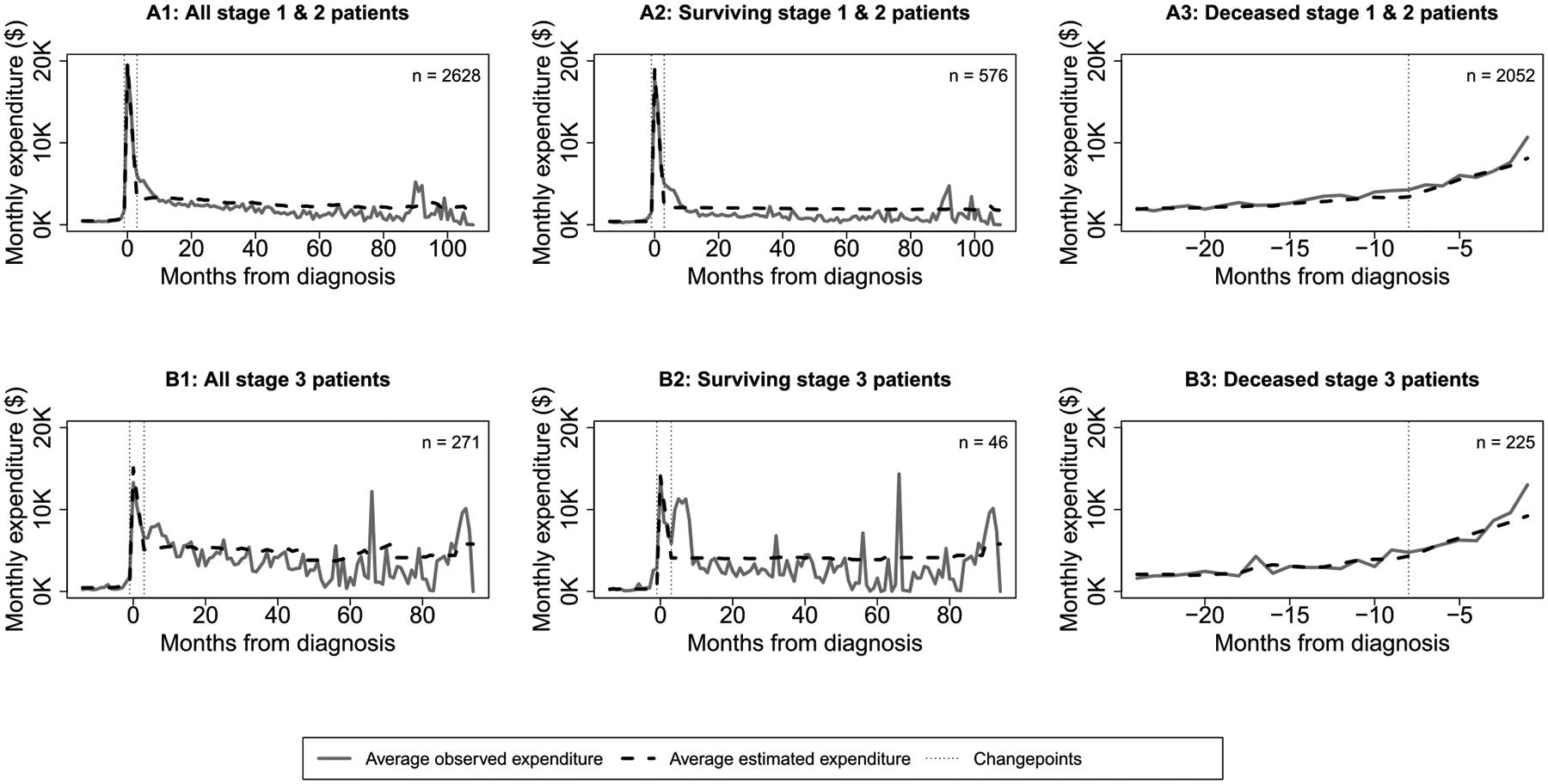
Average observed and estimated monthly expenditure along with change points for different stages of pancreatic cancer patients using the proposed parametric change point approach.

Existing literature suggests that cancer stages are major contributors to healthcare expenditures (see, e.g., Wijeysundera et al., 2012; Tramontano et al., 2019). To further investigate this, we incorporated cancer stage as a categorical variable in our model and stratified the 2,899 pancreatic cancer patients into two groups: the first group consisted of patients with stage I and II pancreatic cancer, and the second group included those with stage III cancer. We then predicted the expenditures for both groups using our model. The expenditure profiles for the stratified groups are shown in Figure 4, Panels A1 and B1. Each group was further divided into cohorts of surviving and deceased patients, as depicted in Panels A2, A3, B2, and B3. The results demonstrate that cancer stage significantly affects expenditure. Specifically, expenditures for stage I and II patients stabilized after the initial treatment phase, whereas stage III patients showed greater variability in costs. This difference is likely attributable to the fact that stage I and II patients often undergo surgical treatment at diagnosis, whereas stage III patients are more commonly treated with chemotherapy, leading to higher medical costs and potentially earlier deaths. Our model effectively captured the first and second change points, as shown in Figure 4 Panels A1, A2, B1 and B2, as well as the final change point before death (see Panel A3 and B3).

The results presented in Table 1 and Figures 3 and 4 demonstrate that our estimation of the change point parameters $\overset{ˆ}{\tau }=\left({\overset{ˆ}{\tau }}_{-1},{\overset{ˆ}{\tau }}_{1},{\overset{ˆ}{\tau }}_{2}\right)=(-1,3,8)$ aligns with the change patterns observed in expenditure profiles. Our method effectively captured the upward and downward trends in these expenditure profiles, with the peaks occurring at diagnosis and stabilizing occurring around three months post-diagnosis. Additionally, the model accurately reflected the increase in expenditures prior to death. Thus, the proposed model has the potential to enhance understanding of expenditure patterns, facilitate planning for costly expenditures, and raise awareness of significant events such as disease diagnosis or impending mortality.

## 4 Discussion

In this project, we introduce a pre-disease phase preceding the diagnosis phase, serving as a baseline for healthcare expenditure, and propose a five-phase piecewise linear model to capture expenditure trajectories and identify transition points between phases. The model uses three population-level parameters to model these transition points, while patient-level characteristics such as demographics, comorbidities, and treatments are incorporated to estimate expenditure amounts and phase durations. Given the change points, we employ penalized generalized estimating equations to estimate the regression coefficients in the proposed model. A grid-search approach is used to estimate the change point parameters by minimizing the residual sum of squares. The innovative aspect of our approach lies in modeling expenditure trajectories using change point detection models, while enhancing accuracy by making the regression parameters dependent on patient characteristics.

We applied this method to estimate expenditure trajectories for stages I–III pancreatic cancer patients using the SEER-Medicare 2005–2014 database, we found that the diagnosis phase initiates one month prior to diagnosis, followed by a three-month initial treatment phase. The stable phase persists until eight months before death, marking the onset of the terminal phase, characterized by increased expenditure once again. The estimation of the change points facilitated precise inference about expenditure patterns for patients with specific diseases. It enhanced our understanding of expenditure pattern dynamics, aiding in more informed decisions regarding treatment funds allocation over time. Moreover, an upward trend in expenditure following a stabilized expenditure period could serve as an early warning sign of deteriorating patient health or disease recurrence.

However, it is important to acknowledge the limitations of the SEER registry data. SEER-Medicare data only captures claims billed to fee-for-service (FFS) Medicare, so individuals enrolled in Part C (Medicare HMO) or without Part B enrollment are likely receiving healthcare that is not recorded in the dataset. In our study, we restricted the analysis to individuals enrolled in both Parts A and B of Medicare, without HMO enrollment during the study period. Otherwise, Medicare expenditures could be misattributed to other insurance and misclassified in the study. For instance, an individual enrolled only in Part C throughout the entire study period would have minimal fee-for-service (FFS) claims, potentially leading to a false perception of zero Medicare expenditure on their care. Such cases must be excluded from the analysis to avoid bias. Therefore, if the analysis were not limited to those with continuous enrollment in Parts A and B but not C during the study, the results would be significantly biased. Since SEER-Medicare users often restrict their analysis to individuals considered “likely to have complete claims” (Enewold et al., 2020), such limitations may affect the generalizability of the findings. More information on the limitations of using SEER registry data can be found at “https://seer.cancer.gov/data-software/documentation/seerstat/nov2022/treatment-limitations-nov2022.html”.

Another limitation of this study is the assumption that the change points are population-specific. This assumption may not hold in heterogeneous populations. For example, patients may receive different treatments, and these treatments could affect the timing of transitions between phases. Additionally, patient characteristics such as age and comorbidities can influence disease progression and, consequently, the change points in expenditure patterns. To address this limitation, a subgroup analysis could be conducted by dividing the data into groups based on treatment types or specific patient characteristics. Applying the proposed model to each subgroup would provide a more tailored understanding of how different factors influence healthcare expenditure trajectories. This approach would offer more nuanced insights into the dynamics of expenditure over time and provide a more comprehensive understanding of how expenditures evolve across different patient subsets, potentially leading to more personalized healthcare strategies.

Despite these limitations, the current article still provides valuable insights into the change points of expenditure trajectories over time.

## Supplementary Material

R Codes for Key Steps of the Case Study

## Figures and Tables

**Table 1:** Distribution of $\overset{ˆ}{\tau }$ based on 1000 bootstrap samples.

${\overset{ˆ}{\tau }}_{-1}$						
Choices	−6	−5	−4	−3	−2	−1
Occurrence %	0%	0%	0%	0%	12%	88%
${\overset{ˆ}{\tau }}_{1}$						
Choices	1	2	3	4	5	6
Occurrence %	0%	2%	82%	16%	0%	0%
${\overset{ˆ}{\tau }}_{2}$						
Choices	5	6	7	8	9	10
Occurrence %	0%	0%	7%	42%	32%	19%

**Table 2:** Estimated values (Est) and their standard errors (SE) of the Γ matrix along with their level of significance (p-value).

$\overset{ˆ}{\beta }$	$\overset{ˆ}{\Gamma }$, SE and p-value of Γ for different covariates									
		Intercept	NCI	Metro/Non-Metro	Grade	Age	Race	Sex	Stage	Treatment
	Ref:			Metro			White	Male	I & II	Chemotherapy
${\overset{ˆ}{\beta }}_{0}$	Est	385.7	262.1	38.8	3.0	47.4	−201.2	113.1	−52.7	−152.2
	SE	156.8	25.2	46.4	8.5	36.5	50.2	36.6	44.1	78.7
	(p-value)	(0.014)	(< 0.001)	(0.403)	(0.724)	(0.194)	(< 0.001)	(0.002)	(0.231)	(0.053)
${\overset{ˆ}{\beta }}_{1}$	Est	6403.8	2283.3	1597.6	−334.4	1058.5	2304.1	−1913.0	55.9	9720.8
	SE	3614.1	345.1	1197.3	147.7	813.9	2448.8	975.6	756.0	1013.3
	(p-value)	(0.076)	(< 0.001)	(0.182)	(0.024)	(0.193)	(0.347)	(0.005)	(0.941)	(< 0.001)
${\overset{ˆ}{\beta }}_{2}$	Est	1426.9	753.7	551.8	−152.9	476.3	790.0	−571.0	−320.4	3740.8
	SE	1240.2	118.9	407.9	50.8	279.2	847.8	336.5	258.4	352.9
	(p-value)	(0.25)	(< 0.001)	(0.176)	(< 0.003)	(0.088)	(0.351)	(0.090)	(0.215)	(< 0.001)
${\overset{ˆ}{\beta }}_{3}$	Est	2508.8	284.3	−19.0	127.3	−323.0	−267.1	−86.9	964.4	−1653.8
	SE	14932.7	1440.4	4914.8	613.4	3363.2	10129.2	4040.7	3125.7	4268.1
	(p-value)	(0.867)	(0.843)	(0.997)	(0.836)	(0.924)	(0.979)	(0.983)	(0.758)	(0.693)
${\overset{ˆ}{\beta }}_{4}$	Est	920.8	5.1	−208.4	20.6	−152.1	154.8	−93.3	−90.8	247.9
	SE	269.1	22.1	72.2	13.0	57.7	121.4	63.6	66.3	91.2
	(p-value)	(0.001)	(0.816)	(0.004)	(0.113)	(0.008)	(0.202)	(0.142)	(0.171)	(0.007)

**Table 3:** Observed and estimated monthly expenditures during the five different phases.

Expenditure phases	Average observed expenditure	Average estimated expenditure
	Mean (SD)	Mean (SD)
Pre-disease phase	450 (993)	523 (431)
Diagnosis phase (1 month)	20702 (36803)	20089 (6273)
Initial treatment phase (3 months)	9835 (13719)	8839 (3067)
Stable phase (varied months)	3312 (5225)	2446 (1061)
Terminal phase (8 months)	5573 (7228)	5577 (2502)
